# Epigenetic Alteration in Colorectal Cancer: Potential Diagnostic and Prognostic Implications

**DOI:** 10.3390/ijms25063358

**Published:** 2024-03-15

**Authors:** Qing Cao, Ye Tian, Zhiyi Deng, Fangfang Yang, Erfei Chen

**Affiliations:** 1 Key Laboratory of Resource Biology and Biotechnology in Western China, Ministry of Education, Northwest University, Xi’an 710069, China; caoqing140508028@163.com (Q.C.); tianye_@stumail.nwu.edu.cn (Y.T.); 2019113145@stumail.nwu.edu.cn (Z.D.); yangfangfang1212@163.com (F.Y.); 2 Provincial Key Laboratory of Biotechnology of Shaanxi Province, Northwest University, Xi’an 710069, China; 3 School of Medicine, Northwest University, Xi’an 710069, China

**Keywords:** colorectal cancer, epigenetics, methylation, ncRNA, biomarkers

## Abstract

Colorectal cancer (CRC), a prevalent malignant tumor of the digestive system, ranks as the third and second in global incidence and mortality, respectively, in 2020, with 1.93 million new cases (≈10% of all cancers). There are 940,000 deaths (≈9.4% of all cancers), and the incidence of CRC in younger patients (under 50 years of age) has become a new trend. The pathogenesis of CRC is primarily attributed to a series of genetic and epigenetic abnormalities within normal colonic epithelial cells, coupled with the reshaping of the tumor microenvironment in the surrounding stroma. This process leads to the transformation of colorectal adenomas into invasive adenocarcinomas. Although genetic changes are known to be the primary driving force in the occurrence and progression of CRC, recent research indicates that epigenetic regulation serves as a crucial molecular marker in cancer, playing a significant role in the pathological and physiological control of interactions between genetics and the environment. This review discusses the current global epidemiology of CRC, its risk factors, and preventive treatment strategies. The current study explores the latest advancements in the epigenetic regulation of CRC, including DNA methylation, histone modifications, and non-coding RNAs (ncRNAs). These developments hold potential as screening tools, prognostic biomarkers, and therapeutic targets for CRC.

## 1. Introduction

Colorectal cancer (CRC) characterized by a high molecular heterogeneity, whose main manifestation is a malignant tumor derived from the glandular epithelium of the colon and rectum, poses a significant threat to human health [1]. In 2020, there were 19.29 million new cancer cases and 9.96 million cancer-related deaths globally. Among males, CRC is the third most common cancer following lung and prostate cancer, with 1.07 million new cases (≈10.6% of all cancers) and approximately 520,000 deaths (≈9.3% of all cancers); among females, CRC ranks as the second most common cancer after breast cancer, with 870,000 new cases (≈9.4% of all cancers) and 420,000 deaths (≈9.5% of all cancers) [2]. Additionally, the mortality rate of late-stage CRC patients under the age of 50 increases by 1.3% annually [3]. The incidence of colon cancer varies by up to 9-fold across different regions worldwide. The highest incidence rates are observed in Europe, Australia/New Zealand, and North America; especially, the highest incidence rates among men and women can be observed in Hungary and Norway. However, in developing countries, such as most areas of Africa and South Asia, the incidence rates of CRC tend to be lower [2]. Notably, China accounted for 24% of the global new cancer-related diagnoses and 30% of cancer-related deaths in 2020. Gastrointestinal cancers occur in 45% of cancer-related deaths in China, a figure significantly higher than in developed countries like the USA and UK. This discrepancy may relate to the more advanced prevalence of early screening and treatment options in developed countries [4]. Despite there being significant advancements in radiotherapy, chemotherapy, and surgical techniques for CRC, existing screening programs and medical technologies are inadequate to curb the high-risk population and continue to expand in developing counties with a vast population base. Multiple risk factors are related to the development of the disease, such as poor lifestyle habits, the inheritance and epigenetic inheritance of related genes, and the development of colorectal adenomas. Whether for individuals, health organizations, or governments, CRC has posed a significant health threat and economic burden challenge. The development of more widely accessible, effective, and non-invasive early screening and diagnostic methods remains an urgent clinical need to detect and remove colon polyps at an early stage.

Epigenetics encompasses the genetic architecture and biochemical modifications of chromatin. In essence, it refers to DNA molecular modifications that can regulate gene activity, leading to heritable phenotypic changes with no need to alter the DNA sequence itself [5]. Compared to extensively researched genetics, the study of epigenetics is a relatively new and emerging area. Nowadays, there are many common epigenetic regulatory mechanisms, such as DNA methylation, chromatin conformational changes, histone modifications, and expression levels of non-coding RNA (ncRNA) alterations. These mechanisms take responsibility for gene expression regulation and epigenetic silencing initiating and maintaining, which are related to a series of intracellular processes, such as cell differentiation, cell division, gene expression, X-chromosome inactivation, embryogenesis, and genomic imprinting [6].

Previous research has shown that epigenetic regulation is one of the fundamental processes for CRC to acquire underlying inherent drug resistance. For instance, aberrant expression of the CRC epigenome may lead to resistance against conventional drugs, such as 5-fluorouracil, oxaliplatin, and cetuximab. RNA sequencing analyses illustrate that approximately 280 non-coding RNA (ncRNA) transcripts can be dysregulated in cetuximab-resistant CRC cells (H508/CR) [7]. Given that epigenetic changes often occur in the early progression of diseases and are involved in nearly all key cancer-related pathways, epigenetic alterations are prime candidates for cancer detection, diagnosis, and prognostic biomarkers. Classic CRC biomarkers, such as mutations in KRAS and BRAF, methylation modifications of NDRG4, BMP3, and SEPT9, high expression of miR-92a and miR-144, and the presence of *F. nucleatum*, are utilized in liquid biopsies for early CRC screening [8,9,10,11,12].

In contrast to irreversible genetic regulation, abnormal epigenetic regulation is reversible with pharmacological interventions. For example, a characteristic of CRC development, EGFR gene amplification, leads to resistance against cetuximab, panitumumab, and necitumumab. However, methylation of histone H3 lysine residues 9 and 27 inhibits EGFR transcription, and treatment with inhibitors targeting KDM4 (a demethylase for H3K9/36) can decrease EGFR amplification, consequently overcoming drug resistance to cetuximab in advanced CRC patients to some extent [13,14]. Additionally, methylation and deacetylation of histone H3 promote the expression of LncRNA-CCAL, which activates the Wnt/β-catenin pathway and subsequently upregulates ABCB1 expression, leading to multidrug resistance (MDR) in CRC [15]. Therefore, understanding the epigenetic drivers associated with CRC pathogenesis and developing specific epigenetic-targeted drugs that target ncRNAs or reverse histone modifications and DNA methylation holds significant potential for precision medicine in CRC patients.

This review gives a concise overview of the current worldwide epidemiology of CRC, with risk factors and strategies for prevention and treatment. It summarizes common types of CRC epigenetic modifications, including DNA methylation, such as the regulation of promoter regions by enhancer methylation, histone modifications, and the regulation by ncRNAs, including microRNAs (miRNAs) and long non-coding RNAs (lncRNAs) as epigenetic regulatory factors. This review screens for potential epigenetic biomarkers from previous articles, aiming to improve early screening, diagnosis, prognosis, and targeted drug treatment for CRC, and lay the foundation for future personalized medicine.

## 2. Colorectal Cancer Risk Factors

Sporadic colorectal cancer (sCRC), which constitutes about 60–65% of CRC cases, lacks a familial genetic history [16]. Adenomas are the typical precursors of CRC, with approximately 85–90% of sCRC developing through the adenoma–carcinoma sequence (ACS) [17]. The malignant transformation of normal colonic epithelial cells into metastatic cancer typically requires decades, providing a substantial period for clinical intervention [18,19]. The accumulation of cancer-driving gene mutations, epigenetic alterations, and environmental interactions are primary factors promoting the development of sCRC [18]. Increasingly, studies have shown that DNA methylation, non-coding RNA regulation, and alterations in apoptosis-regulating and DNA repair genes play crucial roles at different stages of CRC development. Environmental factors such as aging, sedentary lifestyle, hypertension, cirrhosis, family history of colon cancer or inflammatory bowel disease, daily alcohol consumption exceeding one bottle (>600 mL), smoking, obesity, and gut microbiota dysbiosis are high-risk inducers of sCRC [19,20]. Notably, smoking-induced high microsatellite instability, CpG methylation, and BRAF mutations in CRC patients are major preventable causes of cancer deaths [21]. Calcium supplements and adequate consumption of whole grains, fiber, and dairy products seemingly can reduce CRC risk [2].

If patients can be diagnosed in the early stages, their 5-year survival rate can be up to 90% [22]. Although there are multiple treatment options for in situ CRC, aggressive surgery remains the frontline method for curative treatment of CRC; for locally advanced tumors, surgery can alleviate symptoms to some extent and improve prognosis. Clinical surgical treatment is influenced by multiple factors, such as patient age, tumor location, size, and depth of tissue involvement, leading to some patients being unsuitable for surgery or facing the risk of recurrence post-surgery. With advancements in surgical techniques, traditional open surgery has evolved into endoscopic treatments, laparoscopic surgery, and robotic surgery, enhancing the safety and efficacy of surgery. Adjuvant radiotherapy or chemotherapy can significantly reduce tumor volume and prolong survival time. However, the side effects of chemotherapy drugs and patient resistance are pressing issues in clinical treatment. Compared to traditional chemotherapy, targeted drug therapy is more selective to specific tumor targets and can inhibit tumor growth and spread more effectively. For example, drugs that target EGFR or VEGF (Table 1), such as cetuximab, bevacizumab, and panitumumab, have achieved certain success in the treatment of metastatic CRC, but targeted therapy is usually only applicable to specific subtypes of patients, making the body more prone to develop drug resistance. Thus, the future research direction in the development of prognosis-targeted drugs for CRC and the combined application of different targeted drugs in individualized treatment is warranted. In addition, immunotherapy has shown significant efficacy in some CRC patients in recent years. For example, PD-1/PD-L1 antibodies like Pembrolizumab and Nivolumab have yielded notable results. Nonetheless, not all CRC patients can activate their own immune system to attack tumor cells, and individual immune-related side effects vary significantly. In fact, due to the lack of significant symptoms in the early stages of primary local CRC, and the lack of highly sensitive and specific early diagnostic biomarkers, there is a high rate of missed and misdiagnoses. About 50–60% of diagnosed CRC patients eventually progress to late-stage metastatic CRC (mCRC), with hematogenous or lymphatic spread to organs such as the liver, lungs, and bones [23]. The mechanisms driving CRC resistance to various treatments remain unclear, leading to varying degrees of treatment failure for common first-line drugs for CRC (Table 1), with a 5-year survival rate of approximately 14–15% [24,25]. Therefore, identifying reliable biomarkers for early detection of primary and recurrent CRC is crucial for improving patient survival rates.

## 3. Methylation

### 3.1. Mechanism of Methylation Modification

In recent years, increasing evidence has been reported indicating the crucial roles of epigenetic regulatory mechanisms, including DNA methylation, histone modification, and non-coding RNA, in the occurrence, development, and therapeutic processes of CRC.

DNA methylation acts as a kind of stable and inheritable epigenetic modification, playing an important role in the regulation of gene expression in mammals [45]. Being the most extensively studied epigenetic mechanism to date, DNA methylation exhibits various patterns, such as 5-methyl cytosine (5mC), N6-methyladenine (6mA), and 4-methylcytosine (4mC) [46,47]. 6mA and 4mC are predominantly found in the genomes of prokaryotes, while 5mC is the most widespread type of DNA methylation in eukaryotes [48]. The human genome contains approximately 1% of 5mC; at the same time, it is the most studied and understood DNA modification pattern. The 5mC modification occurs within CpG islands where the regions of DNA are rich in CpG dinucleotides. This process includes the transfer of a methyl (CH3-) group from S-adenosyl methionine (SAM) to the fifth carbon position of cytosine, catalyzed by the DNA methyltransferase (DNMT) family, thus forming 5-methylcytosine (5mC). The cytosine-guanine dinucleotides are interconnected by phosphodiester bonds P [49,50].

Approximately 60–70% of gene promoters contain CpG islands. In most normal cells, CpG sites at expressed gene promoters are unmethylated. However, CpG dinucleotides outside of the promoter region often exhibit tissue-specific methylation states, frequently participating in gene transcription regulation [51]. Some studies have illustrated that CpG islands exist in about 40–60% of tumor suppressor gene promoters, typically spanning 200–2000 base pairs (bps). Aberrant methylation of CpG islands in the promoter regions of genes is associated with epigenetic transcriptional silencing of tumor suppressor genes, playing a role in the early stages of CRC regulation [52,53,54]. Methyl-CpG binding domain (MBD) proteins on gene promoters are a key factor in inducing transcriptional silencing through epigenetic changes, impeding the entry of regulatory proteins necessary for active gene transcription. This inhibition directly prevents the binding of transcription factors, ultimately leading to gene transcriptional silencing, However, silenced genes can be reactivated using DNA methylation inhibitors [52]. Additionally, DNA methylation in distal regulatory elements, such as enhancers, is also associated with tissue-specific gene expression regulation [55,56]. However, the presence of 5mC in different types of enhancers is association with promoter interactions, and the necessity for gene expression regulation requires further study. Related studies indicate that enhancer regions exhibit the highest degree of differential methylation regions (eDMRs) during the progression from normal to primary and then metastatic cancers. Abnormal methylation in these regions correlates with gene expression changes in various diseases, including multiple cancers, with eDMRs being most predictive of patient prognosis in metastatic tumors [57]. Hyper-methylation of DNA in genes like IRAK3, CDO1, ADAM2, and SYCP3 is essential for cancer cell survival and growth, while demethylation of these genes leads to cell death and apoptosis. Whole-genome analysis indicates that epigenetic changes in CRC are more frequent than genetic alterations [58]. Therefore, detecting the presence and quantity of CRC-related methylation biomarkers serves as a viable strategy for early monitoring of CRC progression and in vitro targeted therapy of CRC epigenetic regulation. For instance, the sensitivity and specificity of methylated NDRG4 detection for CRC screening are 81% and 92%, respectively [9].

### 3.2. The Role of Methylation in CRC

High methylation of DNA in the promoter regions, which can be commonly observed in CRC leading to gene transcriptional silencing, is considered to be a critical mechanism for tumor suppressor gene inactivation during tumor progression. For example, as the cancer suppressor gene Adenomatous Polyposis Coli (APC) exists in familial CRC and sporadic CRC (sCRC), it plays a key role in regulating the transcriptional activity of β-catenin [59]. The incidence of APC promoter hypermethylation, which is significantly associated with CRC risk, is remarkably higher in colorectal adenomas than in normal colorectal tissues [60]. Furthermore, hypermethylation in promoter regions leads to the inactivation and dysfunction of multiple types of genes, including tumor suppressor genes such as PTCH1 and E-cadherin; DNA repair genes such as MLH1 and MGMT; and apoptosis-related genes such as Apaf1, Bcl2, and p53 [61,62,63,64,65]. Conversely, hypomethylation of DNA may activate oncogene transcription, consequently promoting the development of carcinogenesis and tumor. A potential mechanism is the activation of Long Interspersed Nucleotide Element (LINE-1), which occupies about 18% of the human genome [66]. LINE-1, a non-long terminal repeat retrotransposon, may cause transcriptional disruption and genomic instability. In normal tissues, LINE-1 is typically found to be highly methylated and inactive [67]. Previous research indicates that LINE-1 methylation in CRC adenomas is associated with polyp size and dysplasia severity. Therefore, low methylation and high expression of LINE-1 are conjectured to be associated with negative prognosis in CRC patients, with almost no heterogeneity in LINE-1 methylation within tumors [68,69,70]. Consequently, the combination of the fecal immunochemical test (FIT) with methylation biomarkers and low methylation of LINE-1 may enhance the sensitivity of screening and prognosis in CRC patients.

S-adenosylmethionine (SAM) also exerts its major function as a methyl donor in biological transmethylation processes. The development that SAM can effectively inhibit the hypomethylation of DNA promoters in gastric and CRC has been illustrated by previous studies. Thereby, it can reverse the expression of oncogenes like C-myc and H-ras. This also leads to reduced levels of their mRNA and proteins, subsequently inhibiting the growth of tumor cells [71]. The occurrence of chemotherapeutic resistance increases the recurrence possibility of CRC. The methylation of the CpG island of the hMLH1 gene is related to the resistance to 5-fluorouracil (5-FU). Previous studies have shown that the DNA methyltransferase inhibitor 5-aza-deoxycytidine (5-aza-dC) in the SW480 cell line with high methylation of the hMLH1 promoter induces demethylation of hMLH1. Consequently, this restores the drug sensitivity of SW480 cells to 5-fluorouracil by altering the expression state of the hMLH1 protein [72]. On the other hand, utilizing gene editing technology to selectively reverse aberrant methylation of genes can restore the expression of tumor suppressor genes suppressed by methylation, or selectively silence tumor-promoting genes. For example, Tejedor JR et al. found that promoter methylation silencing of the tumor suppressor gene RSPO2 is a late-stage event in the adenoma-CRC process. Epigenetic reactivation of RSPO2 by dCas9-TET1 fusion protein was associated with significant impairment in cell proliferation in p53^−/−^ cancer cell lines [73]. Therefore, the use of gene editing technology to regulate DNA methylation status has a promising prospect for the treatment of CRC. While still in the research phase, these methods are poised to become integral to future CRC therapies, especially for patients insensitive to conventional treatments, or as adjunctive therapeutic measures.

CRC is a high molecular heterogeneity disease [1]. The colon can be anatomically divided into the left and right halves, which not only have distinct embryonic origins but also differ in pathological types and molecular classifications. Consequently, this results in varied manifestations and clinical responses in patients. For instance, right-sided colon cancer has a higher sensitivity to Syndecan-2 (SDC2) methylation testing. A study based on Chinese fecal samples showed that SDC2 methylation testing had a sensitivity of 81.1% for CRC and a detection rate of 58% for advanced adenomas, leading to missed diagnoses of left-sided colon cancer and adenomas [74]. Research indicates that Tissue Factor Pathway Inhibitor 2 (TFPI2) demonstrates relatively higher sensitivity for left-sided colon cancer. Thus, the newly developed combined methylation status testing of TFPI2 and SDC2 achieves a sensitivity and specificity of 82% and 88.4% for CRC screening, respectively. The sensitivity for advanced adenomas increased to 89.9% with a specificity of 71.6% [75]. Similarly, for the extensively studied blood methylation biomarker SEPT9 in CRC, related studies show that methylated SEPT9 has limited capability in identifying precancerous lesions. However, the positive proportion of SEPT9 is higher in advanced CRC (Stage I: 45%, II: 70%, III: 76%, IV: 79%) and in less differentiated tissues (highly differentiated: 31%, moderately differentiated: 73%, poorly differentiated: 90%), presenting challenges in its clinical applicability [76]. Using a combination of methylated ALX4, SEPT9, and TMEFF2 as biomarkers, the sensitivity for detecting CRC in primary tissue and peripheral blood samples was 84% and 81%, with 87% and 90% specificities, respectively [11]. Further validation is required in larger-scale studies. Currently, many abnormal methylation biomarkers for early CRC detection have been identified in tissue, blood, and fecal samples (Table 2). Specifically, the methylation of vimentin (VIM) is highly expressed in CRC tissues. Fecal DNA testing can identify nearly half (46%) of colon cancer patients with a specificity of 90.0% [77].

## 4. Histone Modification

Histone modifications represent another crucial mechanism of epigenetic regulation. Chromatin, the primary carrier of human genetic information DNA, consists of nucleosomes, the fundamental units made up of an octamer of core histones H2A, H2B, H3, and H4, surrounded by 147 bp of DNA. Research has shown that histone modifications play a significant role in life processes such as gene transcription and DNA damage repair, while aberrant histone modifications are a key regulatory mechanism in many cancer processes, including chemotherapy resistance. A minimum of eight different types of histone modifications have been identified, with common modifications including ubiquitination, phosphorylation, methylation, acetylation, and ADP-ribosylation [106]. Methylation typically occurs when histone methyltransferases (HMTs) catalyze the transfer of a methyl group from S-adenosylmethionine (SAM) to the lysine (Lys) and arginine (Arg) residues on the tails of histones H3 and H4. Histone complexes promote the condensation of genomic DNA, and these post-translational modifications directly influence the expression of oncogenes or tumor suppressor genes by altering the electrostatic charge of the DNA-binding histone tails, or indirectly promoting cancer onset or progression by changing the recognition sites and structures of specific binding proteins [107]. Hence, targeted therapeutic drugs directed at methyltransferases, deacetylases, and related enzymes may become crucial components of future treatments.

Previous research has reported that the expression of H3K9me2 is significantly higher in adenocarcinoma than in normal colonic mucosa. The global dysregulation of H3K9me2 levels is an important epigenetic event in the development and carcinogenesis of colorectal tumors, participating in tumor cell gene regulation through chromatin remodeling [108]. Additionally, the methylation level of H3K27me2 might be an autonomous prognostic factor for asynchronous liver metastasis in CRC [109]. Thus, diffuse H3K9me2 immunopositivity can serve as a useful tool for differentiating tubular adenomas from adenocarcinomas in pathological diagnoses. Furthermore, H3K9me2 could also be a potential therapeutic target for CRC. Similarly, H3K9me3 is specifically elevated in aggressive CRC tissues, with the presence of H3K9me3 positively correlating with lymph node metastasis in CRC patients [110].

Research has identified RGC-32 as a T-lymphocyte cell cycle regulator [111]. In the SW480 cell line, silencing of RGC-32 is related to a reduction in H3K27 trimethylation (H3K27me3), while knockdown of RGC-32 induces an enhancement in acetylation of histone H2B lysine 5 (H2BK5), H2BK15, H3K9, H3K18, and H4K8. Additionally, RGC-32 knockdown significantly increases the proportion of SW480 cells entering the S phase and subsequently the G2/M phase [112]. These data suggest that RGC-32 may promote the development of CRC by regulating the gathering of chromatin core histones.

Evidence suggests that compared to normal colonic tissues, HDAC2 expression is upregulated in CRC tissues, while its expression decreases in metastatic CRC and is associated with poor prognosis in CRC patients [113,114]. The dual role of HDAC2 in the development of CRC may stem from the HDAC2 combination SP1 induction of histone H3K27 deacetylation at the promoter of LncRNA H19, thereby suppressing LncRNA H19 expression. Concurrently, the loss of HDAC2 expression promotes the EMT-mediated CRC lung metastasis process through the LncRNA H19/miR-22-3P/MMP14 axis [114]. On the other hand, histone deacetylase (HDAC) inhibitors have been studied for the treatment of colorectal cancer. These drugs can elevate histone acetylation levels, thereby modulating gene expression. For example, the combination of the histone deacetylase (HDAC) inhibitor vorinostat (VOR) and the autophagy inhibitor chloroquine significantly inhibits the growth and metastasis of tumor cells, and promotes apoptosis in CRC cells by promoting the accumulation of ubiquitinated proteins and the substantial increase in superoxides required for cell death [115]. These findings further support the key role of epigenetic effectors in the development of colorectal cancer.

In addition, technologies such as CRISPR-Cas9 are utilized to modulate DNA methylation status, which can also be applied to regulate histone modification states. By combining editing tools with histone-modifying enzymes or inhibitors, precise control over specific genomic regions can be achieved, thus impacting the survival and proliferation of cancer cells [116]. Overall, histone modification holds significant potential in CRC therapy. With further research in this field and advancements in technology, it is anticipated that more targeted therapeutic strategies involving histone modification will be developed, offering more effective treatment options for CRC patients.

## 5. Non-Coding RNAs

Recent advances in high-throughput sequencing technologies and bioinformatics have revealed that 75% of the human genome is transcribed into RNA, of which only about 2% of transcripts encode proteins. The remaining 98% are ncRNAs, which, until recently, were considered transcriptional “noise” [117,118]. With an increasing number of ncRNAs and their biological functions being identified, it has become evident that ncRNAs play roles as significant as proteins in development, metabolism, and various disease processes. ncRNAs regulate gene expression networks through interactions with other coding or non-coding RNAs, proteins, and DNA. The most extensively studied ncRNAs include small interfering RNAs (siRNAs), circular RNAs (circRNAs), lncRNAs, miRNAs, and the competing endogenous RNA (ceRNA) regulatory networks they form with mRNAs. Identifying specific ncRNAs or proteins bound at chromatin occupancy sites using next-generation sequencing (NGS) and immunoprecipitation techniques has become a crucial method in cancer research, further elucidating the diverse roles of ncRNAs in cancer progression. We will primarily focus on the roles of miRNAs and lncRNAs in the epigenetic regulation of CRC.

### 5.1. MicroRNAs

MicroRNAs, encoded by endogenous genes, contain 18–22 nucleotides as small non-coding RNA molecules. They inhibit mRNA expression to regulate gene expression post-transcriptionally, further modulating the translation level of target proteins which function similarly to siRNAs, and are highly conserved across species. Thanks to the inherent stability of miRNAs in clinical tumor tissue samples and the continuous development in miRNA detection and sequencing technologies, miRNAs have been discovered in bodily fluids such as plasma, saliva, urine, and feces [119]. Increasingly, research shows that genetic deletions or amplifications at miRNA genomic loci, epigenetic methylation, and transcription factor-mediated primary miRNA regulation always alter miRNA expression in cancer. This alteration leads to overexpression, amplification, or loss of epigenetic silencing of miRNAs targeting one or multiple tumor suppressor genes, inhibiting anti-tumor pathways. Even miRNA mutations can lessen or eradicate binding to key targets, thereby generating new mRNA sequences and modifying critical growth balance regulators [120,121]. Previous research indicates that miRNAs control the expression of about 30% of essential human genes vital for normal survival and development, hence dysregulated miRNA expression is involved in the pathogenesis of many cancers [122].

Particularly in the carcinogenic process of CRC, there are approximately 1900 miRNA encoding genes in the human genome, with 250 miRNAs experiencing changes in the richness of expressions or roles in CRC, covering different stages from initiation to progression and metastasis [123]. For instance, the downregulation of miR-143 activates the RAS-RAF-MEK pathway, targeting the mRNA translation of the oncogene KRAS, and inhibiting CRC growth [124]. miR-21, one of the most commonly upregulated miRNAs in inflammation processes and CRC development, is also a genetic and pharmacological regulatory target for multiple diseases. Studies have shown that a lack of miR-21 leads to lowered Ki67 expression in CAC mouse tumors, weakened tumor cell proliferation, increased epithelial marker E-cadherin, and decreased β-catenin and SOX9. Moreover, a lack of miR-21 reduces STAT3 and Bcl-2 activation, leading to increased apoptosis in CAC mouse tumor cells [125]. miR-21 can also cause CRC or colitis-associated cancer (CAC) by the PI3K/AKT, PDCD4/TNF-α, and IL-6/STAT3 signaling networks, activating the tumor cell invasion/metastasis process [126]. In addition, the high expression level of miR-21 in feces can significantly distinguish between CRC tumors, lymph nodes, and metastatic stages III–IV and I–II, with a sensitivity and specificity of 88.1% and 81.6%, respectively [127]. The APC gene, a tumor suppressor in CRC, with somatic mutations in 80% of sporadic CRC presents adenomatous polyposis coli (APC) at an early stage of colorectal tumors [128]. miR-135 directly targets the 3′ untranslated region of APC, suppressing APC expression and inducing downstream Wnt pathway activity [129]. Furthermore, fecal miR-135b-5p serves as a non-invasive diagnostic biomarker for late-stage CRC, useful for diagnosing patients with CRC at TNM stages III/IV. An increasing number of miRNAs are being used as diagnostic (Table 3) and prognostic (Table 4) biomarkers for CRC.

Recent advancements highlight that within different cancer types, individual miRNAs have a multitude of gene targets, acting as either tumor suppressors or oncogenes, depending on their targets. This complexity underpins the intricate regulation of gene expression and cancer progression. For instance, miR-429 acts as a tumor suppressor in renal cell carcinoma (RCC), breast cancer (BC), gastric cancer (GC), glioblastoma (GBM), esophageal cancer (EC), osteosarcoma oral squamous cell carcinoma (OSCC), cervical cancer (CC), pancreatic cancer, tongue squamous cell carcinoma (TSCC), nephroblastoma, nasopharyngeal carcinoma (NPC), and soft tissue sarcoma. Conversely, miR-429 promotes tumor progression in endometrial cancer (EmCa), prostate cancer (CaP), and lung cancer (LC). However, miR-429 exhibits contradictory roles in CRC, hepatocellular carcinoma (HCC), bladder cancer, and ovarian cancer (OC), with its function varying along with the tumor’s developmental stages [130]. In CRC progression and metastasis, overexpression of miR-429 enhances proliferation and migration in HT29 and HCT116 cells, while its downregulation inhibits proliferation and migration in LOVO cells both in vitro and in vivo. Mechanistically, miR-429 promotes CRC progression and metastasis by directly targeting HOXA5 [131]. In terms of drug treatment, overexpression of miR-429 correlates positively with adverse reactions to 5-FU chemotherapy in CRC patients [130]. Moreover, research by Hong Liu et al. suggests that berberine (BER) and evodiamine (EVO) could become promising anti-tumor drugs for CRC treatment by downregulating miR-429 expression [132].

Currently, the standard first-line chemotherapy for metastatic colorectal cancer (mCRC) includes 5-FU combined with oxaliplatin or cetuximab. Most anti-cancer therapies intend to induce apoptosis in cancer cells to improve survival rates for CRC patients. For example, miR-129 promotes apoptosis in CRC cells and enhances sensitivity to 5-fluorouracil by inhibiting Bcl-2 [133]. miR-34a inhibits macrophage activation by targeting the key effector SMAD4 in the TGF-β signaling pathway, mediating resistance to oxaliplatin in CRC cells [134]. Studies have identified miR-106a as one of the miRNAs with the most significant expression differences between 5-FU responsive and non-responsive CRC patient plasma samples [135]. The overexpression of miR-106a reduced sensitivity to 5-FU in HCT116 and SW620 cells, whereas miR-106a antagonists enhanced their drug sensitivity. Mechanistically, miR-106a is negatively correlated with DUSP2 in CRC tumor samples, and its elevation increases COX-2 and stem cell maintenance gene (SOX2 and OCT4) expression levels [136]. On the other hand, DUSP2 expression is significantly reduced in many human cancers, inversely correlating with malignancy severity. The downregulation of DUSP2 induces carcinogenesis in CRC cells and increases CRC resistance to cetuximab [137]. Thus, miR-106a and DUSP2 may serve as potential targets in CRC patients’ response to 5-FU and cetuximab treatment.

Furthermore, miRNA expression in CRC is regulated by DNA methylation and histone modifications. Nearly 10% of miRNAs in CRC cells are controlled by DNA methylation [138]. MiR-34 members, acting as tumor suppressors, target TP53, LEF1, CDK4, CCNE2, SMAD4, and MYC [139,140]. Compared to normal tissues, hypermethylation of the miR-34 promoter leads to the downregulation of miR-34a and miR-34c in human CRC tissues. Hence, methylation of miR-34b/c is associated with CRC metastasis and invasion. Difluorinated curcumin (CDF) may act as a novel demethylating agent, reviving the expression of the miR-34 family, and thereby becoming a new drug for treating CRC [141]. Similarly, hypermethylation of the tumor suppressor miR-133b promoter significantly downregulates its expression in human CRC tissues. miR-133b enhances CRC cells’ chemosensitivity to anti-tumor drugs 5-fluorouracil (5-FU) or vincristine (VCR) by directly downregulating ABCC1 [142]. Therefore, miR-133b could serve as a prospective sensitizer for drug-resistant CRC.

**Table 3:** A catalogue of abnormal miRNA diagnostic biomarkers.

Diagnostic Markers	Specimens	Epigenetic Changes	Sensibility (%)	Specificity (%)	Reference
miR-92a-1	serum	up-regulated miRNAs	81.8	95.6	[143]
miR-29a + miR-92a	plasma	up-regulated miRNAs	83	84.7	[144]
miR-92	plasma	up-regulated miRNAs	89	70	[145]
miR-28-3p + miR-106a-5p + miR-542-5p + let-7e-5p	plasma	up-regulated miRNAs	99.7	90.9	[146]
miR-135b-5p	serum	up-regulated miRNAs	93.1	72.7	[147]
miR-21	serum	up-regulated miRNAs	86.05	72.97	[127]
miR-21	serum	up-regulated miRNAs	82.8	90.6	[148]
miR-21	saliva	up-regulated miRNAs	97	91	[149]
miR-1246+ miR-1268b + miR-4648	serum	up-regulated miRNAs	50.7	90.2	[150]
miR-106a	tissue	up-regulated miRNAs	53	85	[151]
miR-106b	serum	up-regulated miRNAs	85.2	78	[152]
miR-429	tissue	up-regulated miRNAs	71.79	62.82	[153]
miR-200c + miR-18a	plasma	up-regulated miRNAs	84.6	75.6	[154]
miR-223 + miR-92a	plasma	up-regulated miRNAs	76.3	68.8	[155]
miR-424-5p	serum	up-regulated miRNAs	79	72.6	[156]
miR-375	plasma	down-regulated miRNAs	76.92	64.63	[157]
miR-145	tissue	down-regulated miRNAs	90	88	[158]
miR-23b	tissue	down-regulated miRNAs	78	70	[158]
miR-195	tissue	down-regulated miRNAs	72	68	[158]
miR-24	plasma	down-regulated miRNAs	78.38	83.85	[159]
miR-320a	plasma	down-regulated miRNAs	92.79	73.08	[159]
miR-423-5p	plasma	down-regulated miRNAs	91.89	70.77	[159]
mi-24 + mi-320a + mi-423-5p	plasma	down-regulated miRNAs	92.79	70.77	[159]
miR-143-3p	serum	down-regulated miRNAs	61.3	74.2	[156]
miR-135b-5p	stool	up-regulated miRNAs	96.5	74.1	[147]
miR-21	stool	up-regulated miRNAs	86.05	81.08	[127]
miR-92a	stool	up-regulated miRNAs	89.7	51.7	[12]
miR-144	stool	up-regulated miRNAs	78.6	66.7	[12]
miR-92a + miR-144	stool	up-regulated miRNAs	96.6	37.9	[12]
miR-223 + miR-92a	stool	up-regulated miRNAs	73.9	82.2	[155]
miR-20a	stool	up-regulated miRNAs	55	82	[160]
miR-221	stool	up-regulated miRNAs	62	74	[161]
miR-18a	stool	up-regulated miRNAs	61	69	[161]
miR-221 + miR-18a	stool	up-regulated miRNAs	66	75	[161]
miR-29a	stool	down-regulated miRNAs	85	61	[162]
miR-224	stool	down-regulated miRNAs	75	63	[162]

**Table 4:** A catalogue of abnormal miRNA prognostic biomarkers.

Prognostic Markers	Specimen	Epigenetic Changes	CRC Staging	Reference
miR-21	tissues	up-regulated	adenomas/carcinomas	[163]
miR-92a	tissues	up-regulated	adenomas/carcinomas	[164]
miR-25	tissues	up-regulated	advanced (III-IV)/lymph node metastasis/distant metastasis	[165]
miR-1246, miR-1268b, miR-4648	serum	up-regulated	stage II and III/recurrence	[150]
miR-1260b	tissues	up-regulated	lymph node metastasis and venous invasion	[166]
miR-141	plasma	up-regulated	advanced colon cancer	[167]
miR-429	tissues	up-regulated	5-FU treatment	[153]
miR-29a	tissues	up-regulated	stage II CRC/recurrence	[168]
miR-29a	serum	up-regulated	liver metastatic	[169]
miR-34a	plasma	up-regulated	adenoma	[170]
miR-106b	serum	up-regulated	lymph node metastasis and distant metastasis	[152]
miR-135b-5p	serum, stool	up-regulated	stage III and IV	[147]
miR-126	serum	down-regulated	early-stage liver-metastatic	[171]
miR-429	tissues	down-regulated	stage III and IV/lymphatic metastasis	[172]
miR-24, miR-320a, and miR-423-5p	plasma	down-regulated	postoperative metastasis	[159]

### 5.2. LncRNA

Non-coding RNAs longer than 200 nucleotides are categorized as long non-coding RNAs (lncRNAs), functioning as signals, decoys, guides, and scaffolds [173]. The specific functions of lncRNAs are closely related to their subcellular localization. In the nucleus, lncRNAs regulate chromatin remodeling and transcription; in the cytoplasm, they act as ceRNAs, regulating mRNA translation and degradation by targeting microRNA response elements (MREs), and are involved in regulating gene expression and interfering with post-translational modifications in various cancers, including CRC [174,175,176]. Dysregulation of lncRNAs in CRC often results in oncogenic or tumor-suppressive functions. For example, lncRNA DLEU1 promotes CRC cell proliferation and migration by recruiting the NURF chromatin remodeling complex subunit SMARCA1 to the promoter of KPNA3 [177]. Similarly, high expression levels of LINC01094, H19, and MALAT1 are closely associated with metastasis and poor prognosis in CRC patients [178,179,180]. Therefore, regulation of lncRNA expression offers potential biomarkers and therapeutic targets for CRC treatment (Table 5).

Research by Gao R et al. demonstrated that lncRNA CASC15 is upregulated in oxaliplatin-resistant CRC tissues and cells, correlating with poor prognosis. Silencing the competitive endogenous RNA CASC15 to regulate the miR-145/ABCC1 axis overcomes resistance to oxaliplatin in CRC [181]. Moreover, long non-coding RNA CRART16, acting as a ceRNA, confers 5-FU resistance to CRC cells by inhibiting miR-193b-5p and regulating HMGA2 expression, activating the MAPK signaling pathway [182]. Additionally, overexpression of CRART16 increases the proportion of CD44+/CD133+ cells and may promote resistance to cetuximab in CRC cells through the miR-371a-5p/ERBB3/MAPK pathway [183]. Furthermore, lncRNA LIFR-AS1, as a competitive endogenous RNA for miR-29a, inhibits its expression, upregulates downstream target TNFAIP3, and regulates resistance to PDT in CRC [184]. Consistently, downregulated lncRNA CBR3-AS1 potentially phagocytose mature miR-29a in the cytoplasm of CRC cells, consequently inhibiting miR-29a-mediated cell migration and invasion [185]. Meanwhile, increased expression of miR-29a, by directly targeting KLF4, regulates E-cadherin and matrix metalloproteinase 2, promoting CRC metastasis [186]. This suggests the potential of CBR3-AS1 and miR-29a inhibitors as novel anti-metastatic drugs for CRC.

FGD5-AS1, a newly discovered lncRNA with a length of 3772 nucleotides, is abnormally overexpressed in various cancer tissues and closely associated with lymph node metastasis, tumor invasion, survival time, and recurrence rate [187]. In the progression of CRC, lncRNA FGD5-AS1 upregulates CDCA7 expression by competitively inhibiting miR-302e, promoting the proliferation, migration, and invasion capabilities of CRC cells [188]. Therefore, FGD5-AS1 serves as a potential diagnostic or prognostic marker for various cancers.

Moreover, lncRNAs are significant regulators of DNA methylation, especially in cancer. During rapid tumor growth, hypoxia stimulates neovascularization through vascular endothelial growth factor (VEGF), essential for tumor survival. Studies have shown that promoter methylation of EGFL7 induces a silencing of miR-126; treatment with 5-aza-CdR restores miR-126 expression, with VEGF as a direct target of miR-126, leading to decreased VEGF expression and inhibiting CRC tumor invasion and angiogenesis to some extent [189]. Similarly, hypomethylation of the oncogene LINC00460 promotes metastasis in CRC cells [190]. Thus, miR-126 and LINC00460 may serve as potential therapeutic targets in CRC. Additionally, recent studies have indicated that lncRNA HOTAIR mediates the mutual regulation between histone-lysine N-methyltransferase (EZH2) and DNA methyltransferase (DNMT1) [191]. Some small molecules, like AC1Q3QWB (AQB), can selectively block the HOTAIR-EZH2 interaction, offering a novel approach to cancer treatment [192]. LncRNAs can also regulate genomic DNA methylation through DNMT. For example, as a competitive endogenous RNA, lncRNA HIF1A-AS2 target adsorbs miR-129-5p, indirectly promoting the expression of DNA methyltransferase 3 alpha (DNMT3A), finally facilitating EMT and CRC progression [193].

Recent studies have shown that the dysregulation of intestinal flora is associated with incidence of CRC. Hong J et al. found that high glycolysis of gut microbes is associated with poor prognosis in patients with colorectal cancer. *F. nucleatum* activates transcription of lncRNA ENO1-IT1 by raising transcription factor SP1 binding efficiency to the ENO1-IT1 promoter region. Elevated ENO1-IT1, serving as a KAT7 histone acetyltransferase instructional module, alters histone modifications on the target gene ENO1, promoting glycolysis and tumorigenesis in CRC [194]. In addition to this, gut microbial metabolites such as short chain fatty acids (SCFAs) also have an important impact on the occurrence and progression of CRC. Alvandi et al. found that 70.4% of high-risk CRC individuals exhibited significantly decreased concentrations of acetate, propionate, butyrate, or total SCFAs in their feces, while 66.7% of CRC patients had significantly lower concentrations of acetate and butyrate in their feces compared to healthy controls [195]. Mowat C et al. further found that gut microbial SCFA or compounds mimicking their effects could serve as a promising therapeutic avenue for CRC by enhancing anti-tumor immune function in CRC patients [196]. The potential mechanism is that SCFAs function as histone deacetylase (HDAC) inhibitors, modulating the intestinal inflammatory response by blocking cell cycle progression and promoting the induction of apoptosis, which ultimately reduces the proliferation of tumor cells, thus exerting a positive effect on colorectal cancer treatment [197]. Besides directly acting on intestinal epithelial cells, SCFAs also play critical anti-inflammatory roles in regulating local and systemic immune cells by promoting the production of antimicrobial compounds, inhibiting neutrophils and macrophages, activating regulatory T cells, and inducing dendritic cell tolerance [198,199]. Thus, the antitumor effects of SCFAs may involve more complex mechanisms beyond the tumor cells themselves. Therefore, screening or targeting the modulation of intestinal microbiota metabolites and specific epigenetic mechanisms also represents emerging therapeutic strategies for diagnosing and treating CRC.

**Table 5:** A catalogue of abnormal lncRNAs biomarkers.

LncRNAs Diagnostic Role	LncRNAs Prognostic Role	References
H19, MALAT1, CCAT1, LEF1-AS1, PVT1, LINC01410, RP11-296E3.2, HIF1A-AS1, NRIR	LINC01094, MALAT1, CACS15, CRART16, CBR3-AS1, FGD5-AS1, LEF1-AS1, LINC00460, HIF1A-AS2, LINC00114, HOTAIR, LINC00261, PVT1, LINC01410, RP11-296E3.2	[178,179,180,181,182,185,187,190,193,200,201,202,203,204,205,206]

## 6. Summary and Future Perspective

Colonoscopy is still the most reliable method for CRC screening and diagnosis. Previous studies show that colonoscopy can reduce the incidence of CRC by 69% and mortality by 68% [207]. However, due to its invasive nature, which can cause discomfort to patients, along with being expensive, time-consuming, and potentially leading to complications such as infections, especially in elderly patients, its acceptability and widespread adoption, particularly in developing countries, is limited. CT colonography, a non-invasive imaging method using computed tomography (CT) and specialized software to generate high-resolution images of the colon, is more convenient than colonoscopy but cannot obtain tissue samples, thus necessitating colonoscopy upon finding abnormalities. Common blood biomarkers like carcinoembryonic antigen (CEA) and carbohydrate antigen 19-9 (CA19-9), though non-invasive, have good specificity for detecting occult CRC but due to other conditions that can also elevate their expression, their sensitivity is only about 40% to 60%. Therefore, more than half of CRC patients could be misdiagnosed based on CEA or CA19-9 alone, limiting their clinical screening utility [208,209]. Cheng H et al. found that combining miR-141 with CEA could further improve the accuracy of detecting distant metastasis in colon cancer [167].

Although the fecal occult blood test (FOBT), the first step in CRC screening programs, cannot directly diagnose CRC, it can help screen for potential issues, reducing mortality in 15% to 33% of CRC patients [210]. Despite possible false positives due to exercise, diet, and medication factors, FOBT remains an effective, widely applicable method for screening high-risk CRC populations due to its simplicity and low cost. Fecal immunochemical tests (FITs) are more effective than traditional FOBT for large-scale CRC screening, detecting minor bleeding from abnormalities like polyps or tumors in the colorectal or rectal regions, thus can identify twice as many CRC patients as FOBT, and are particularly effective for early-stage CRC screening [211]. However, an FIT has limited sensitivity for CRC stage I and matured adenomas, and the instability of specific antibodies used in FITs during transport and storage can lead to false-negative results, posing challenges for widespread screening [212].

Furthermore, DNA in fecal samples can be tested to detect genetic and epigenetic DNA changes in tumor cells shed into feces. As a relatively new screening method, it offers non-invasive and convenient advantages. Therefore, real-time monitoring of the disease status of patients can be achieved, so as to adjust the treatment plan individually. Ahlquist et al. conducted CRC screening using combined target genes in fecal samples, such as APC, K-ras, p53, and vimentin, achieving a positive detection rate of up to 46% for adenomas ≥ 1 cm, significantly higher than FOBT [213]. Multi-target fecal DNA testing also shows higher detection rates for advanced adenomas and serrated polyps larger than 1cm compared to an FIT [214]. While fecal DNA testing can study cfDNA from colon tumors, it may yield higher false-positive rates due to free DNA from other gastrointestinal parts, and a mixture of heterogeneous DNA from various fungi, bacteria, and gut microbiota diversity; moreover, miRNA levels in fecal samples are lower than in tumor tissues or blood, posing potential limitations in sensitivity and specificity compared to other CRC screening methods, hence being still under research and development.

In summary, all conventional screening methods have limitations. Blood biomarkers, easily obtained non-invasively and stored, represent a promising approach for early CRC diagnosis. Integrating other high-sensitivity complementary markers into a multi-omics approach could further improve the diagnostic efficacy for advanced polyps, precancerous lesions, and early CRC.

**Figure 1:**
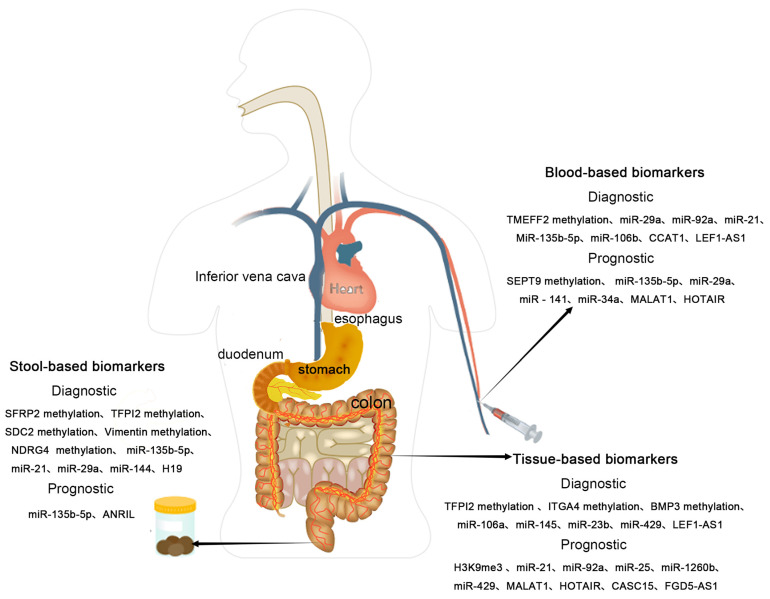
Epigenetic biomarkers in CRC.

This review summarizes common epigenetic biomarkers for CRC (see Figure 1), including the methylation of VIM, NDRG4, BMP3, SDC2, SEPT2, SEPT9 genes, and the high expression of miR-21, miR-135b-5p, miR-92a-1, ANRIL [215]. However, their clinical diagnostic results for early CRC screening and prognosis still show individual variability. This variability might be due to CRC’s inherent heterogeneity, different diets and lifestyles, and various molecular tumor subtypes leading to significant specificity and sensitivity differences in the same epigenetic marker; moreover, limited sample sizes in most biomarker screenings and the lack of validation in large, independent patient cohorts with unclear control group definitions and detailed CRC population information like age, gender, tumor stage, and location necessitate further large-scale clinical data support before the widespread application of hypermethylated DNA and miRNA as clinical biomarkers for CRC detection and prognosis. Therefore, comparing and conclusively interpreting different study outcomes for the same molecular marker remain challenging.

Moreover, epigenetic changes offer potential reversibility through drug treatment, with the types and numbers of available epigenetic modifications steadily increasing. However, clinical safety evidence for epigenetic modification drugs and significant survival benefits for CRC patients treated with such drugs remain scarce. Overall, epigenetic biomarkers hold strong potential for CRC screening, diagnosis, and drug treatment targets, but their sensitivity, specificity, and security still require further exploration.

## Figures and Tables

**Table 1:** Common drugs and therapeutic targets for colorectal cancer.

Drug Name	Target Gene	Affected Colorectal Cancer Staging	Reference
Pertuzumab	HER2	Metastatic	[26]
Encorafenib	BRAF	Metastatic	[27]
Ipilimumab	CTLA-4	Metastatic, MSI-H/dMMR	[28]
5-FU	DNA synthesis and repair	Metastatic	[29]
Cetuximab	EGFR	Metastatic	[30,31]
Panitumumab	EGFR	Metastatic	[32,33]
Trastuzumab	HER2	Metastatic	[26,34]
Trametinib	MEK1, MEK2	RAS and RAF mutation	[35]
Dostarlimab	PD-1	Metastatic, MSI-H/dMMR	[36]
Nivolumab	PD-1	Metastatic, MSI-H/dMMR	[37]
Pembrolizumab	PD-1	Metastatic, MSI-H/dMMR	[38,39]
Olaparib	PARP1/2	BRCA mutation	[40]
Bevacizumab	VEGF-A	Metastatic	[41,42]
Ziv-aflibercept	VEGF-A, VEGF-B, IGF-1	Metastatic	[43]
Regorafenib	VEGFR2, TIE2, PDGFR, FGFR, KIT, RET, BRAF, BRAFV600E	Metastatic	[44]

MSI-H/dMMR: microsatellite instability-high/deficient mismatch repair.

**Table 2:** A catalogue of abnormal methylation biomarkers in colorectal cancer.

Diagnostic Markers	Specimen	CRC Staging	Epigenetic Changes	Sensibility (%)	Specificity (%)	Reference
(1)						
ALX4	serum	early-stage CRC	methylation	46.6 (21/45)	66.3 (11/16)	[78]
ALX4	plasma	early-stage CRC	methylation	47.8 (87/182)	93.5 (159/170)	[11]
CDH1	serum	early-stage CRC	hypermethylation	18 (3/17)	100 (10/10)	[79]
NEUROG1	serum	early-stage CRC	methylation	55.5 (25/45)	81.3 (13/16)	[78]
P16	serum	early-stage CRC	hypermethylation	71 (12/17)	100 (10/10)	[79]
RASSF1A	serum	early-stage CRC	hypermethylation	24 (4/17)	100 (10/10)	[79]
RASSF1A	plasma	early-stage CRC	methylation	93 (28/30)	53 (16/30)	[80]
RUNX3	serum	early-stage CRC	promoter hypermethylation	65 (11/17)	100 (10/10)	[79]
TFPI2	serum	early-stage CRC	methylation	18 (39/215)	100 (20/20)	[81]
TFPI2	stool	early-stage CRC	methylation	76 (50/66)	93 (28/30)	[82]
TFPI2	tissue	early-stage CRC	methylation	99 (114/115)	94 (45/48)	[82]
SDC2, TFPI2	stool	early-stage CRC	methylation	82 (237/289)	88.4 (192/217)	[75]
SDC2	tissue	early-stage CRC	methylation	96.8 (120/124)	ns	[74]
SDC2	stool	early-stage CRC	methylation	81.1 (159/196)	93.3 (167/179)	[74]
TMEFF2	plasma	early-stage CRC	methylation	70.9 (129/182)	95.2 (162/170)	[11]
c9orf50, twist1, kcnj12, znf132	plasma	early-stage CRC	methylation	80 (140/175)	97 (54/56)	[83]
EFHD1	plasma	early-stage CRC	promoter methylation	79 (19/24)	78 (75/96)	[84]
BMP3	plasma	early-stage CRC	methylation	75 (44/59)	70 (26/37)	[10]
BMP3	tissue	early-stage CRC	methylation	81 (24/30)	ns	[10]
C9orf50	tissue	early-stage CRC	methylation	60	80.6	[85]
SFMBT2	tissue	early-stage CRC	methylation	85.7	87	[85]
ITGA4	tissue	early-stage CRC	methylation	85.7	87	[85]
THBD	tissue	early-stage CRC	methylation	84.1	87	[85]
ZNF304	tissue	early-stage CRC	methylation	70	100	[85]
SFMBT2, ITGA4, THBD, ZNF304	tissue	early-stage CRC	methylation	96.1	87	[85]
RARB2, p16INK4a, MGMT, APC	tissue	early-stage CRC	promoter methylation	77 (20/26)	100 (20/20)	[86]
RARB2, p16INK4a, MGMT, APC	stool	early-stage CRC	promoter methylation	62 (16/26)	100 (20/20)	[86]
AGTR1, WNT2, SLIT2	stool	early-stage CRC	methylation	78.1 (50/64)	89.5 (34/38)	[87]
(2)						
SDC2	serum	TNM I-IV	methylation	87.0 (114/131)	95.2 (119/125)	[88]
SEPT9	plasma	TNM I-IV	methylation	61.8 (76/123)	89.6 (112/125)	[89]
SEPT9	plasma	TNM I-IV	methylation	74.7 (136/182)	96.5 (164/170)	[11]
SEPT9	plasma	TNM I-IV	methylation	50.9 (27/53)	91.4 (1331/1457)	[90]
SEPT9	plasma	TNM I-IV	methylation	74.8 (101/135)	87.4 (298/341)	[91]
SEPT9	tissue	TNM I-IV	methylation	78 (99/127)	97 (116/120)	[11]
NDRG4	stool	TNM I-IV	promoter methylation	61 (17/28)	93.3 (42/45)	[92]
NDRG4	tissue	TNM I-IV	methylation	81 (68/84)	92 (77/84)	[9]
NDRG4	blood	TNM I-IV	methylation	54.8 (46/84)	78.1 (66/84)	[9]
NDRG4	urine	TNM I-IV	methylation	72.6 (61/84)	85 (71/84)	[9]
NDRG4	stool	TNM I-IV	methylation	76.2 (64/84)	89.1 (75/84)	[9]
OSMR	tissue	TNM I-IV	promoter methylation	80 (80/100)	4 (4/100)	[93]
OSMR	stool	TNM I-IV	promoter methylation	38 (26/69)	95 (77/81)	[93]
SFRP1	plasma	TNM I-IV	promoter methylation	80 (20/25)	92 (33/36)	[94]
PHACTR3	stool	TNM I-IV	methylation	66 (29/44)	100 (30/30)	[95]
NEUROG1	serum	UICC I-II	methylation	61 (59/97)	91	[78]
SFRP2	serum	TNM I-IV	methylation	66.9 (113/169)	93.7 (59/63)	[96]
SFRP2	stool	TNM I-IV	hypermethylation	94.2 (49/52)	95.2 (23/24)	[97]
SFRP2	stool	TNM I-IV	methylation	84 (142/169)	54 (34/63)	[96]
SFRP2	tissue	TNM I-IV	methylation	88.2 (149/169)	34.9 (22/63)	[96]
SPG20	stool	TNM I-IV	hypermethylation	80.2 (77/96)	100 (30/30)	[98]
HLTF	serum	TNM I-IV	hypermethylation	32.7 (16/49)	92.7 (38/41)	[99]
hMLH1	serum	TNM I-IV	hypermethylation	42.9 (21/49)	97.6 (40/41)	[99]
MGMT	stool	TNM I-IV	methylation	48.1 (25/52)	100 (24/24)	[100]
vimentin	serum	TNM I-IV	methylation	31.1 (14/45)	62.5 (10/16)	[78]
vimentin	stool	TNM I-IV	methylation	45.7 (43/94)	90.0 (178/198)	[77]
vimentin	stool	TNM I-IV	methylation	72.5 (29/40)	86.9 (106/122)	[101]
vimentin	urine	TNM I-IV	hypermethylation	75 (15/20)	90 (18/20)	[102]
APC	serum	TNM I-IV	hypermethylation	6.1 (3/49)	100 (41/41)	[99]
Wif-1	plasma	TNM I-II	methylation	36.7 (89/243)	90.6 (250/276)	[103]
APC, MLH1, HLTF	serum	TNM I-IV	promoter hypermethylation	57.1 (28/49)	90.2 (37/41)	[99]
APC, MGMT, RASSF2A, Wif-1	plasma	TNM I-II	methylation	86.5 (210/243)	92.1 (253/276)	[103]
RASSF1A, SFRP2	stool	TNM I-IV	promoter methylation	75.0 (63/84)	89.4 (101/113)	[104]
MGMT, MLH1, VIM	stool	TNM I-IV	promoter methylation	75.0 (45/60)	86.5 (32/37)	[105]
ALX4, SEPT9, TMEFF2	plasma	TNM I-IV	promoter methylation	81 (147/182)	90 (153/170)	[11]
ALX4, SEPT9, TMEFF2	tissue	TNM I-IV	promoter methylation	84 (107/127)	87 (105/120)	[11]

Note: The above table is for the detection of colorectal cancer sensitivity and specificity data in healthy individuals. ns: not specified.
